# The development of sympathy from 5 to 7 years: increase, decline or stability? A longitudinal study

**DOI:** 10.3389/fpsyg.2014.00468

**Published:** 2014-05-20

**Authors:** Jutta Kienbaum

**Affiliations:** Institut für Psychologie, Pädagogische Hochschule KarlsruheKarlsruhe, Germany

**Keywords:** sympathy, empathy, concern, prosocial behavior, childhood

## Abstract

In the present study the development of sympathy in a group of 85 children (43 girls) was investigated over a 3-year period, starting with the last year of child care, when the children were 5 years-old. Sympathy was measured via different measures: two standardized observations, where the children were observed as they witnessed the distress of a puppet in two different situations; two follow-up interviews with the children immediately after the observations; a self-report questionnaire and two other-report questionnaires by parents and teachers. At all three periods the observations and the children’s self-reports (interviews, questionnaire) were intercorrelated. The teachers’ and the parents’ reports were not significantly correlated with any of the other measures at time 1. At times 2 and 3, a few low but significant correlations emerged. As a consequence, the other reports were dropped from further analyses and a composed sympathy measure consisting of observations and self-reports was created. Rank-order stability of this composed measure over the course of the 3 years proved to be high; suggesting that interindividual differences maintained stability. Mean-level differences showed a significant increase over the course of the study with the highest increase in the initial 2 years. Neither gender nor the interaction between gender and time were significant. In conclusion, the measurement of sympathy has proven valid for the childrens’ observations and self-reports. To the question of age-correlated development, stability in sympathy is firstly high and secondly sympathy increases mainly during the time between the last year in child care and the first year in elementary school.

## INTRODUCTION

The ability to sympathize with another person’s pain or distress may be the most important bases for interpersonal relationships. Knowledge about the development of this ability in children is therefore crucial for our understanding of human social interaction, especially for the motivation of prosocial behavior (Paulus, 2014). Advances in the understanding of the relations between sympathy and prosocial behavior have been obtained in part by a conceptual differentiation between terms like sympathy, empathy, personal distress, perspective taking, etc., (Batson, 1991; Bischof-Köhler, 2012; Eisenberg et al., 2014).

Sympathy has been defined by Eisenberg et al. (2007) as “an emotional response stemming from the apprehension of another’s emotional state or condition, that is not the same as the other’s state or condition but consists of feelings of sorrow or concern for the other” (p. 546). Empathy, in contrast, is defined by Bischof-Köhler (2012) as “a process in which an observer vicariously shares the emotion or intention of another person and thereby understands what this other person feels or intends” (p. 41). In this sense, being empathetic means to be aware that the source of the shared emotion is in the other person. Thus, empathy should not be confused with emotional contagion, a state that “…occurs when the vocal, facial and gestural cues of one individual generate a similar state in the perceiver” (Decety and Svetlova, 2012, p. 8) – like, for example, contagious laughter or mass panic. Empathy can occur not only as a reaction to another person’s mishap, but also to positive emotions like joy (Singer, 2006; Light et al., 2009; Sallquist et al., 2009). It is value-neutral (Eisenberg et al., 2014).

The motivational consequences of empathy to other’s negative emotions can be quite different, sympathy is but one possibility. Another possible reaction is distress, which means that for example a child is more self- than other-focused and experiences feelings of tension (Batson, 1991). Finally, empathy can also lead to schadenfreude – a feeling that could not be enjoyed without empathy (Bischof-Köhler 2012; Schulz et al., 2013).

A final point concerning definition is the relation between sympathy and prosocial behavior. Sympathy is not necessarily related to prosocial behaviors, since behaviors like sharing or donating can, for example, also be motivated by social norms. Yet relations with altruistic behavior such as comforting are found frequently, so that comforting behavior is often used as an indicator for sympathy (Kienbaum, 2001; Eisenberg et al., 2014).

The first empathy-like phenomenon that can be observed in development is the so-called reactive newborn cry. Newborns start to cry as soon as they hear another baby cry (Simmer, 1971; Sagi and Hoffman, 1976; Dondi et al., 1999). This is not yet empathy because of course the newborns lack the awareness that the feeling originates in another baby and not in themselves; it’s an example of emotional contagion.

When does empathy appear for the first time? What is required for its full-blown experience is the self-other distinction of subjective experience. This ability to distinguish between self and other emerges during the second year of life and is usually measured by children’s ability to recognize themselves in a mirror (Rouge Test, Amsterdam, 1972). The middle of the second year of life, when self-recognition usually occurs, is also the time when the first sympathetic-comforting actions in children have been reported (e.g., Zahn-Waxler et al., 1992; Svetlova et al., 2010).

In order to test empirically whether self-recognition is a prerequisite of compassionate behavior, Bischof-Köhler (2012) conducted a series of investigations with more than 120 children between 16 and 24 months. She found that only children who recognized themselves in the mirror showed concern and compassion toward a sad playmate. Yet not all recognizers showed sympathy, so it was concluded that self-recognition is a necessary but not sufficient precondition for empathy (but see , for less clear relations). In a recent publication by Roth-Hanania et al. (2011), the idea that this is the time when sympathy first occurs has been challenged. The authors investigated the responses of 37 infants from 8 to 16 months to the distress of their mother and a videotaped peer. “Concerned affect” was operationalized mainly in terms of sadness in face or voice and appeared in 8- and 10-months-olds already, an age when the above described self-other distinction cannot be assumed. Prosocial behavior occurred very seldom in this age group; self-recognition was not assessed. Whether these results can be interpreted in terms of an earlier onset of other-oriented empathic responding or whether the sad vocal and facial reactions of the infants would better be interpreted as a form of emotional contagion is open to discussion at this point; of course more empirical evidence with children younger than 1 year has to be accumulated before any generalizations can be made (Davidov et al., 2013).

Is there a normative development of sympathy? The most popular theory concerning this question has been formulated by Hoffman (2000). He postulates an age-correlated increase of sympathy brought about by progress in social-cognitive development. According to him, individuals make progress “…as they grow up in understanding the causes, consequences, and correlates of an increasingly complex array of emotions” (p. 80). Hoffman describes five “stages” in the development of sympathy. Four of them take place in infancy; beginning with the already mentioned “reactive newborn cry” and ending in the middle of the second year of life, when the children realize that the other has inner states independent of their own (called “veridical empathic distress” by Hoffman). The fifth and last stage pertains already to school-age-children, who are able to experience sympathy beyond the immediate situation; for example they can feel sympathy for an entire group that is not present (e.g., “poor children”) and realize that the lives of others may be generally sad or happy.

Although Hoffman does not offer his own empirical data to test this theory, there is some empirical support for his assumptions. Yet, the vast majority of research has been conducted in the infant years. For example, the twin studies by Knafo et al. (2008), and Zahn-Waxler et al. (2001) found longitudinally an increase of empathic concern and prosocial behavior between 14 and 36 months and so confirmed a gradual transition from strong self-concern to empathic concern for others in early childhood.

In contrast to Hoffman’s position, Hay (1994) proposes a decline in prosocial reactions from infancy to childhood because, for example, children learn rules about who deserves help, and so “…prosocial behavior becomes less of a general social impulse and more of a considered decision” (p. 38). Volland et al. (2004) found support for the effect of the postulated rules: 4- to 8-year-old children were more willing to offer help to another child if her damage was high, if she was not to blame for it, if the child was younger and familiar and if she had also helped before. The importance of these attributes increased with increasing age of the children. Although Hay’s theory pertains to prosocial behavior, it can easily be applied to the development of sympathy as well, since statements like “it’s his own fault” clearly indicate that this person does not deserve our sympathy. For example, van der Mark et al. (2002) found an increase of empathic concern for the mother’s distress from 16 to 22 months in girls, but a decrease for the distress of a stranger, thus supporting the rule that a familiar person is more worthy of sympathy than an unfamiliar one.

Recently, a third position was outlined by Davidov et al. (2013). These authors propose that empathic concern may not grow over time at all because “…it cannot be assumed that the affective core of empathy qualitatively changes with age” (p. 129). Empirical support for this assumption comes from studies that did not find age-correlated increases in sympathy, like Light et al. (2009) in a cross-sectional study with 6- and 10-year-old children, Vaish et al. (2009) with a cross-sectional comparison of toddlers aged 18 and 25 months and Volbrecht et al. (2007) with a longitudinal study of nearly the same age group (19–25 months).

Whereas many of the studies cited so far investigated infants or toddlers, empirical evidence especially in terms of longitudinal data about the development of sympathy during the *childhood* years is sparse. Eisenberg and Fabes (1998), cited in Eisenberg et al. (2014) conducted a meta-analysis using studies published from 1983 until about 1996 and “…found an age-related increase in empathy and sympathy across childhood and adolescence, at least for observational and self-report indices (but not for solely facial or physiological indices). However, they did not examine when in childhood the age-related changes were most evident” (p. 187). Since then, only a few longitudinal studies have addressed the question of age-related changes in sympathy during childhood. Hastings et al. (2000) examined concern for others in children from ages 5 to 7. Observable concern was stable for children at low or moderate risk of clinical behavior problems, but decreased significantly for children at high risk. Malti et al. (2013) investigated a sample of Swiss children at 6, 7, and 9 years of age. 47% of the children reported increasing sympathy over time, 43% stayed stable on a high level and 10% reported consistently low levels of sympathy over the course of the study. In general, self-reported sympathy increased between 6 and 7 as well as between 7 and 8 years (Tina Malti, e-mail message to author, February 5, 2014).

Hence, there is empirical support for each of the three positions outlined above: increase, decrease, and no changes of sympathy with age. One severe problem in comparing and evaluating the different studies is that most of them rely on only one single measure, either self-report or observation, thus limiting the explanatory power of the results. Any single measure is only a partial assessment of the underlying construct, and at the same time it incorporates error and bias. What is urgently needed (apart from a new meta-analysis) is longitudinal data that relies on multiple methods from multiple sources. A welcome supplement effect of such a study would be that information on the methods validity could be gathered by investigating whether they converge or not.

Another aspect concerning development is that despite possible age-related changes in children, there is the possibility that interindividual differences emerge in early childhood and stay stable thereafter. The existence of an altruistic personality has been debated for many years mainly in the social psychological, but also in the developmental literature (Eisenberg et al., 1999; Knafo et al., 2008; Paulus et al., 2013). According to Eisenberg et al. (2006), there is evidence of modest stability among measures of prosocial or empathy-related responding. For example, Hastings et al. (2000) found evidence of modest stability between observed concern for others at 5 and 7 years. Malti and Buchmann (2010) report modest stability for self- and other sympathy reports within informant (child, mother, and teacher) from 6 to 7 years. More longitudinal data, based on multiple methods from multiple sources is needed to make sure that we can generalize the conclusion that interindividual differences tend to stay stable from the preschool-years onward.

Finally, methods also play a role in the question of gender differences. Sympathy is a gender-sensitive topic; it is a widely held view that females are more sympathetic than males. Yet, the empirical evidence is mixed: the largest divergences favoring girls have been found for self- and other-report measures, whereas only few differences occurred in studies using physiological responses to evocative stimuli (Eisenberg et al., 2014). In a recent meta-analytic review on gender differences in emotion expression in children, Chaplin and Aldao (2013) found a small effect size for girls showing more sympathy expressions than boys. In the present study, several methods will be used with the same sample over a period of 3 years. Thus, it can be tested whether gender differences are method-dependent and whether this pattern changes with age or stays the same across the whole time period.

The goal of the present study was to assess the developmental trajectories of sympathy in middle childhood in a three-wave longitudinal study, using a multi-method multi-informant approach including observations in standardized situations, different types of self-reports and reports by mothers and teachers. Specifically, it was examined

(a) whether the different methods would converge or not, so that conclusions about their validity could be drawn,
(b) whether there would be a significant increase in sympathy, as hypothesized by Hoffman (2000), or a decrease, as postulated by Hay (1994) or no changes (Davidov et al., 2013), and
(c) whether interindividual differences in sympathy would be stable over the 3 year period of the study.

## MATERIALS AND METHODS

### PARTICIPANTS

The data were collected in South Tyrol, a rural, touristy area in the mountains of northern Italy, where the majority of the population speaks German as their first and Italian as their second language. South Tyrol has a comparatively low level of unemployment (about 3% in 2011). The capital Bozen-Bolzano is the biggest town with about 100.000 inhabitants (Autonomous Province of South Tyrol, 2013).

Data collection started in 2009 with 85 children (43 girls, *M* = 70.25 months or 5.85 years, *SD* = 3.79 months). Out of these, 12 (14%) visited a child care center in Bozen-Bolzano, the rest attended child care centers in and around Brixen-Bressanone, a small town with about 20.000 inhabitants. In 2010, one girl moved; the mean age of the remaining 84 children was *M* = 79.58 months or 6.67 years (*SD* = 3.77 months). In 2011, 83 children (41 girls, 42 boys), with a mean age of *M* = 91.75 months or 7.6 years (*SD* = 3.83 months) remained in the study. Consent was received from school authorities and parents.

Mothers (*N* = 76 at T1, *N* = 77 at T2 and T3), 33 child care teachers and 31 first and second grade teachers completed questionnaires concerning the children’s dispositional sympathy (see below).

The children were mostly from middle-class families. 70 mothers and 67 fathers provided information about their highest educational achievement. Of the mothers, 30% reported that they had completed high school, followed in frequency by the completition of university (27%), vocational training (27%), middle school (9%), and others (7%). Concerning the fathers, 36% reported that they had completed a vocational training, followed in frequency by the completition of university (33%), high school (21%), middle school (9%), and others (1%).

Information concerning siblings was available for 73 of the participating children; of these, 44 (60%) had one sibling, 18 (25%) had two siblings, one had three siblings (1%) and 10 (14%) had no siblings.

### PROCEDURES AND MEASUREMENTS

Sympathy was measured via the following methods:

(a) two standardized observations, where the children were observed when they witnessed the distress of a puppet in two different situations;
(b) two follow-up interviews with the children immediately after the observations;
(c) a German version of the child-report sympathy scale (Zhou et al., 2003);
(d) a German version of the parents’ and the teachers’ reports of children’s sympathy (Zhou et al., 2003).

During the *observational trials*, the children were videotaped as they witnessed the distress of a puppet in two different situations (Kienbaum et al., 2001). The puppet was about 60 cm tall and was controlled by a trained student.

In the first situation, called “sadness,” at T1 the child and puppet played with two balloons that had been blown up ahead of time and then watched a short film together. During the film, the puppet’s balloon bursts and the puppet “cries” for 30 s, followed by 30 s in which there is a gradual subsiding of the distress.

In the second situation, called “pain,” at T1 the child and puppet were sitting together drawing pictures. When the puppet decided to stand up and get some new coloring pencils, it bumped into a chair and feigned injury for 30 s, followed again by 30 s in which there was a gradual subsiding of the distress.

The observations took place in a separate room in the child care center or school; the order was counterbalanced. There was a minimum of one day between the two observations. The reactions of the child were videotaped by two cameras and coded by two trained, independent observers, each on a scale from 0 (does not occur at all) to 5 (very strong). The criteria used for the evaluations were similar to those used by Eisenberg et al. (1988, p. 303) as well as those used in other research groups (Kienbaum and Trommsdorff, 1997). The behavior of a child was labeled as “sympathetic-comforting” when she interrupted her activity, softened her face, oriented her attention toward the puppet by looking at it, talked to it in a soft comforting voice and/or caressed the puppet or offered her own balloon.

Interrater reliabilities were established for the whole sample by means of Cohen’s weighted kappas^1^ (Cohen, 1968). Discrepancies between ratings were decided in conference. The final rating was the conferenced rating. The resulting values were *k*_w_ = 0.92/“pain” and *k*_w_ = 0.91/“sadness” at T1, *k*_w_ = 0.92/“pain” and *k*_w_ = 0.95/“sadness” at T2 and *k*_w_ = 0.82/“pain” and *k*_w_ = 0.80/“sadness” at T3, all *p* < 0.001.

The simulations for pain and sadness were different every year. At T2, the puppet simulated pain when a big book fell on her leg. The simulation of sadness took place when the doll wanted to paint a picture with water colors and the water flowed over her image. At T3, the puppet simulated pain when she bumped her head while she tried to pick up a puzzle piece from the ground. She simulated sadness after she had “accidentally” torn a picture with an animal photo.

Shortly after the observations, the children were *interviewed*. The puppet yawned and “went to sleep,” whereupon the student proposed to clean up the room together with the child. Meanwhile, the student asked what had happened and why the puppet cried. Finally, the child was asked if she felt sorry for the puppet and if so, how much on a scale from 1 (not at all) to 3 (very much).

On a different day, we interviewed the children using the five positively formulated items from the child-report sympathy scale (Zhou et al., 2003; e. g. “I often feel sorry for other children who are sad or in trouble”). Items were translated into German and read aloud. If the children answered that they felt sorry, they were asked how much (a little bit or a lot; 1 = do not feel sorry; 3 = do feel sorry a lot). Cronbach’s alphas from T1 to T3 were 0.82, 0.84, and 0.69.

Finally, two questionnaires – the *Parents’ Reports on Children’s Sympathy* and the *Teachers’ Reports on Children’s Sympathy* (Zhou et al., 2003) – were administered to the children’s parents and teachers, respectively. Out of the five items, only those four that were positively formulated were used for all further analyses, since the negatively formulated item lowered reliability. Four items remained (e.g., “My child/this child usually feel sorry for other children who are upset or sad”; 1 = child is not sympathetic, 3 = child is very sympathetic). Items were translated into German. Cronbach’s alpha for the mothers from T1 to T3 were 0.67, 0.78, and 0.80. For the teachers, the corresponding values were 0.86, 0.92, and 0.93.

Observations of and interviews with the children took place either in the child care center (T1) or the school (T2 and T3) in a quiet, separate room. The parents’ questionnaires were handed out to the children with an envelope to be sent back. Child care teachers and school teachers were given the questionnaires in the institution.

## RESULTS

In the following, descriptive analyses for the different measures of sympathy are presented first. Secondly, intercorrelations at the three time intervals are presented. Finally, the results from the rank-order stability analyses (correlations) and the mean-level stability analyses are presented.

Means and standard deviations for the different measures are depicted in **Table 1**.

**Table 1:** Means and standard deviations of methods measuring sympathy.

	*M (SD)*		
	T1	T2	T3
Observation sadness	1.71 (2.11)	1.96 (1.92)	1.96 (1.74)
Girls	1.67 (2.14)	1.97 (1.88)	2.18 (1.64)
Boys	1.76 (2.10)	1.95 (1.99)	1.73 (1.83)
Observation pain	0.89 (1.50)^ab^	1.36 (1.80)^a^	1.53 (1.79)^b^
Girls	0.76 (1.32)	1.17 (1.72)	1.59 (1.75)
Boys	1.02 (1.68)	1.55 (1.88)	1.48 (1.85)
Follow-up interview sadness	2.10 (0.79)^c^	2.25 (0.67)	2.40 (0.59)^c^
Girls	2.17 (0.76)	2.26 (0.66)	2.49 (0.51)
Boys	2.03 (0.82)	2.24 (0.69)	2.32 (0.64)
Follow-up interview pain	2.05 (0.80)^de^	2.37 (0.70)^d^	2.43 (0.55)^e^
Girls	1.91 (0.72)	2.40 (0.68)	2.46 (0.50)
Boys	2.18 (0.86)	2.34 (0.72)	2.40 (0.60)
Child-report sympathy scale	2.10 (0.60)^fg^	2.44 (0.52)^f^	2.40 (0.43)^g^
Girls	2.14 (0.60)	2.56 (0.46)	2.48 (0.39)
Boys	2.05 (0.60)	2.32 (0.55)	2.33 (0.45)
Parents’ reports of children’s sympathy	2.43 (0.45)^h^	2.47 (0.47)	2.59 (0.44)^h^
Girls	2.56 (0.37)	2.60 (0.43)	2.76 (0.29)
Boys	2.30 (0.47)	2.35 (0.49)	2.42 (0.50)
Teachers’ reports of children’s sympathy	2.18 (0.61)	2.27 (0.63)	2.26 (0.64)
Girls	2.46 (0.48)	2.50 (0.54)	2.52 (0.56)
Boys	1.90 (0.62)	2.04 (0.64)	2.00 (0.62)

*Whole sample sizes range from 77 to 84 subjects due to missing values. ^abcd…^ Values in a row marked with the same characters differ significantly (*p* < 0.05). Observations scales were from 0 to 5; all other scales from 1 to 3.*

As can be seen in **Table 1**, sympathy either increased or stayed stable. Repeated measures ANOVAs were run for each method with the three times as the within-subjects factor and the gender of the child as the between-subjects factor in order to test whether differences in values were significant or not. For three of the methods – the follow-up interview pain, the child-report sympathy scale and the teachers’ reports of children’s sympathy scale – the assumption of sphericity had been violated, therefore the degrees of freedom were corrected using the Greenhouse–Geisser estimate of sphericity. Omega squared (ω^2^), a correction of η-squared, is reported as measure of effect size, since it is a population estimate and less biased than η-squared (Field, 2009).

Concerning the *observation sadness*, neither the main effect of time, *F*(2,156) = 0.97, *ns*, ω^2^ = 0.00, nor gender, *F*(1,78) = 0.12, *ns*, ω^2^ = 0.00, nor the interaction between gender and time, *F*(2,156) = 0.95, *ns*, ω^2^ = 0.00, proved significant. For the *observation pain*, the main effect of time was significant, *F*(2,162) = 7.12, *p* < .001, ω^2^ = 0.02, whereas the main effect of gender, *F*(1,81) = 0.32, *ns*, ω^2^ = 0.00, and the interaction between gender and time, *F*(2,162) = 1.05, *ns*, ω^2^ = 0.00, were not. Regarding the *follow-up interview sadness*, again the main effect of time proved significant, *F*(2,142) = 7.12, *p* < 0.001, ω^2^ = 0.03, whereas the main effect of gender, *F*(1,71) = 0.77, *ns*, ω^2^ = 0.00, and the interaction between gender and time, *F*(2,142) = 0.50, *ns*, ω^2^ = 0.00, were not. As to the *follow-up interview pain*, the main effect of time was significant, *F*(1.81,128.81) = 11.84, *p* < 0.001, ω^2^ = 0.06, in contrast to the main effect of gender, *F*(1,71) = 0.16, *ns*, ω^2^ = 0.00, and the interaction between gender and time, *F*(1.81,128.81) = 2.60, *ns*, ω^2^ = 0.00. For the *child-report sympathy scale*, the main effect of time was highly significant, *F*(1.83,147.82) = 17.00, *p* < 0.001, ω^2^ = 0.08, whereas the main effect of gender just fell short of significance, *F*(1,81) = 3.58, *p* < 0.07, ω^2^ = 0.02. The interaction between gender and time was not significant, *F*(1.83,147.82) = 0.74, *ns*, ω^2^ = 0.00. For the *parents’ reports of children’s sympathy scale*, both main effects of time, *F*(2,136) = 3.85, *p* < 0.05, ω^2^ = 0.02, and gender, *F*(1,68) = 11.48, *p* < 0.001, ω^2^ = 0.10, were significant, whereas the interaction between gender and time was not, *F*(2,136) = 0.44, *ns*, ω^2^ = 0.00. Finally, for the *teachers’ reports of children’s sympathy scale*, the main effect of time was not significant, *F*(1.48,115.48) = 0.85, *ns*, ω^2^ = 0.00, whereas the main effect of gender was highly significant, *F*(1,78) = 27.10, *p* < 0.001, ω^2^ = 0.19. The interaction between gender and time was not significant, *F*(1.48,115.48) = 0.20, *ns,* ω^2^ = 0.00.

In sum, there was no significant interaction between time and gender in any of the methods. For two of the methods, time had no significant main effects (observation sadness, teachers’ reports); whereas in all the other methods, values of children’s sympathy increased with increasing age. Results of the *post hoc* Bonferroni tests are shown in **Table 1**.

Concerning the two observations, sympathetic reactions were significantly higher in the simulation of sadness as compared to the simulation of pain at all three times [*t*(81) = -3.45, *p* < 0.001, *t*(83) = -3.56, *p* < 0.001 and *t*(82) = -2.43, *p* < 0.05 at T1, T2 and T3, respectively).

Next, the gender differences were inspected more closely. *Post hoc* Bonferroni tests revealed that at all three time intervals, mothers and teachers rated girls as more sympathetic than boys (*ps* < 0.05 for T1 and T2 and *p* < 0.001 at T3 for the maternal ratings and *ps* < 0.001 at T1, T2, and T3 for the teacher ratings). Concerning the other five methods, only one single difference emerged: at time 2, girls described themselves as more sympathetic on the child-report sympathy scale (*p* < 0.05).

In order to test the validity of the different measures, their intercorrelations were computed at the three intervals in a next step; results can be seen in **Tables 2–4**.

**Table 2:** Intercorrelations of the methods measuring sympathy T1.

Spearman correlation coefficient, one-tailed						
	2	3	4	5	6	7
1. Observation sadness	0.38***	0.29**	0.24*	0.25*	0.02	-0.11
2. Observation pain	–	0.10	0.21*	0.12	-0.04	0.06
3. Follow-up interview sadness		–	0.74***	0.44***	0.06	0.07
4. Follow-up interview pain			–	0.48***	-0.03	0.10
5. Child-report sympathy scale				–	0.05	0.14
6. Teachers’ reports of children’s sympathy					–	0.17
7. Parents’ reports of children’s sympathy						–

Sample sizes range from 70 to 85 subjects due to missing values; *p < 0.05, **p < 0.01, ***p < 0.001.

**Table 3:** Intercorrelations of the methods measuring sympathy T2.

Spearman correlation coefficient, one-tailed						
	2	3	4	5	6	7
1. Observation sadness	0.74***	0.51**	0.33**	0.35**	0.16	0.10
2. Observation pain	–	0.52***	0.45***	0.25*	0.06	0.23*
3. Follow-up interview sadness		–	0.70***	0.61***	-0.01	0.08
4. Follow-up interview pain			–	0.63***	-0.07	0.20*
5. Child-report sympathy scale				–	0.02	0.18
6. Teachers’ reports of children’s sympathy					–	-0.03
7. Parents’ reports of children’s sympathy						–

Samples sizes range from 77 to 84 subjects due to missing values; *p < 0.05, **p < 0.01, ***p < 0.001.

**Table 4:** Intercorrelations of the methods measuring sympathy T3.

Spearman correlation coefficient, one-tailed						
	2	3	4	5	6	7
1. Observation sadness	0.68***	0.43***	0.46***	0.40***	0.18*	0.34**
2. Observation pain	–	0.40***	0.48***	0.43***	0.12	0.41***
3. Follow-up interview sadness		–	0.64***	0.51***	0.06	0.29**
4. Follow-up interview pain			–	0.55***	0.10	0.31**
5. Child-report sympathy scale				–	0.20*	0.21*
6. Teachers’ reports of children’s sympathy					–	0.27*
7. Parents’ reports of children’s sympathy						–

Sample sizes range from 71 to 83 subjects due to missing values; *p < 0.05, **p < 0.01, ***p < 0.001.

The correlations showed quite a clear pattern: at time 1, the observations and the children’s self-reports were intercorrelated, whereas the parents’ and teachers’ reports were not significantly correlated with any of the other measures. The same held true for time 2, although parents’ reports were significantly correlated for at least two of the measures. At time 3, the pattern is the same, but at this interval the parents’ rating also significantly correlated with all the other measures, though coefficients were not as high as for the other correlations.

In a next step, the rank-order was examined with correlations for the three different time points. Results are shown in **Table 5**.

**Table 5:** Rank-order stability of methods measuring sympathy.

Spearman correlation coefficient, one-tailed			
	T1–T2	T1–T3	T2–T3
Observation sadness	0.56***	0.55***	0.61***
Observation pain	0.36***	0.43***	0.65***
Follow-up interview sadness	0.49***	0.48***	0.53***
Follow-up interview pain	0.42***	0.45***	0.59***
Child-report sympathy scale	0.45***	0.26**	0.49***
Parents’ reports of children’s sympathy	0.45***	0.47***	0.53***
Teachers’ reports of children’s sympathy	0.21*	0.33**	0.78***

Samples sizes range from 72 to 84 subjects due to missing values; *p < 0.05, **p < 0.01, ***p < 0.001.

Since the reports from the mothers, child care and elementary school teachers did not correlate continuously with the other methods, they were dropped from all further analyses. The remaining five methods (observations and self-report-measures of sympathy) were standardized and aggregated; the means and standard deviations at T1, T2, and T3 are depicted in **Table 6**. Afterward, a repeated measurement ANOVA with the three intervals as the within-subjects factor and the gender of the child as the between-subjects factor was computed. A significant effect of time emerged, *F*(2,162) = 23.95, *p* < 0.001, ω^2^ = 0.05, whereas neither gender, *F*(1,81) = 0.08, *ns*, ω^2^ = 0.00, nor the interaction between gender and time, *F*(2,162) = 2.03, *ns*, ω^2^ = 0.00, turned out to be significant. *Post hoc* Bonferroni tests showed that the difference between time 1 and the two later time intervals was significant (both *p*s < 0.001).

**Table 6:** Means and standard deviations of aggregated sympathy.

	*M (SD)*		
	T1	T2	T3
Total sample	2.09 (1.26)	2.65 (1.30)	2.78 (1.17)
Girls	2.03 (1.16)	2.65 (1.23)	2.93 (1.03)
Boys	2.14 (1.36)	2.63 (1.38)	2.63 (1.29)

*Total sample sizes are *N* = 85 at T1, *N* = 84 at T2 and *N* = 83 at T3.*

The rank-order of the aggregated measure was computed again by means of the Spearman’s rank correlation coefficient; the resulting values were *r* = 0.65, *p* < 0.001 for T1–T2, *r* = 0.63, *p* < 0.001 for T1–T3 and *r* = 0.73, *p* < 0.001 for T2–T3.

## DISCUSSION

The aim of the present study was to examine the mean-level change and rank-order stability of sympathy during middle childhood in a three-wave longitudinal study, using a multi-method multi-informant approach including observations in standardized situations, different types of self-reports and reports by mothers and teachers. There was evidence of rank-order stability and mean-level change in nearly all of the methods.

To begin with, mean level change over the study’s 3 years run appeared in one of the standardized observations (“pain”), both follow-up interviews, the child-report sympathy scale and the parents’ reports of children’s sympathy scale. The means of the teachers’ reports of children’s sympathy scale were quite high and stayed stable over the course of the study. The means of the sadness-simulation were significantly higher as opposed to the pain-simulation and also stayed stable during the 3 years of assessment. Thus, in five of the seven different methods that were administered, there was an increase in sympathy. A decrease was not observed at all, and stability occurred only in two of the methods. In sum, this pattern of results gives support to the theory by Hoffman (2000) where an increase in empathic responding over the childhood years is assumed.

But before generalizing these results, the validity of the different measures was assessed by computing intercorrelations between them in every year. Here, a very clear pattern emerged: the observations and self-reports were significantly intercorrelated at all three times, whereas ratings of teachers and parents did not correlate with any of the other methods. It seems as if parents and teachers had difficulties in rating children’s sympathy correctly. Social desirability might play a role here, as well as, the fact that sympathetic reactions are not so frequently observed, since often adults intervene very quickly when they witness a mishap (Caplan and Hay, 1989). Interestingly, in the second year of the study, a few small, significant correlations between the parent’s ratings and the other methods emerged. In the third year, the parent’s ratings even correlated significantly with all the other methods, though the coefficients were still modest in size. Maybe the parents became more aware of the phenomenon of sympathy during the 3 year period of the study, and started to observe their children more carefully and so their rating became better over time. Thus, parental ratings may be useful, but only after the topic of interest has occupied their minds for some time.

The ratings by parents and teachers are not only conspicuous with regard to the correlations with other methods, but also with regard to gender differences. With the exception of the child-report sympathy scale at T2, parents and teachers were the only ones who rated girls continuously higher in sympathy than boys. Apparently, their ratings are highly influenced by gender stereotypes, what creates further doubt to the validity of their assessments (see also Malti and Buchmann, 2010, for gender differences in teacher’s ratings). Gender differences in other reported sympathy may reflect adult’s conceptions of what boys and girls are supposed to be like rather than how they actually behave. The fact that these ratings stayed stable over the 3 years course of the study shows how deeply rooted these stereotypes are.

In sum, the observations and the self-reports of the children were closely interrelated and so renders the conclusion that they are valid. This is particularly interesting with regard to the observations, since it was a puppet that simulated the distress and the pain, what of course gives rise to the question whether reactions to the mishap of a puppet can be generalized to “real people.” The results of this study suggest an affirmative answer, because children’s descriptions of their sympathy and their reactions to the mishap of the puppet were in accordance. Unlike adults, children apparently perceive these “living puppets” as real playmates and do not separate sharply between the worlds of imagination and reality. This interpretation is supported by the fact that only very few children (one in 2009, three in 2010, and two in 2011) said in the follow-up interview that “this was just a puppet.”

The difference in mean values between the two simulations of pain and sadness replicated earlier findings (Kienbaum et al., 2001). Apparently, the simulation of pain seems to be the more “difficult” situation for the children, at least for the younger ones. Pain might be a state for them that cannot be taken away easily, whereas in the case of the bursting balloon there are more options for comforting the sad playmate. Interestingly, the difference between the two simulations became smaller in the third year of the study, due to the increasing means in the pain-simulations, whereas the mean values in the sadness-simulations stayed stable. This is remarkable since the means of both situations were always below the median of the scale, in contrast to the self- and other reports where children in average always scored higher than the median. Thus, the verbal methods produce higher sympathy scores as opposed to the observations. Social desirability might play a role in this context, since being sympathetic may be part of a positive self-portrayal that extends also to ones children.

Concerning all the methods, rank-order stability was quite high. The only exception was the correlation between the child care teachers (T1) and the school teachers (T2 and T3); this is not surprising since different persons were involved. Apart from that, there were numerous significant, positive relations across time within methods. Correlations were highest for the aggregated measure of sympathy. Thus, considerable evidence for differential stability over the course of the 3 years of the study was obtained, supporting the idea of an overall sympathy disposition. Apparently, the so called “altruistic” personality tends to develop quite early, even before the entry into school, and is highly consistent over time. The reasons for this stability are probably due to a number of factors, which include both genetic contributions and continuity of socialization influences. Concerning the genetic contributions, the already mentioned twin studies obtained evidence of heritability of empathy-related responding (Zahn-Waxler et al., 2001; Knafo et al., 2008). Furthermore, there is evidence that sympathy is linked to temperamental traits like inhibition (e.g., van der Mark et al., 2002), that likely have a constitutional basis (but are also influenced by the environment; Kienbaum et al., 2001). As to socialization, continuity in the childrearing environment like a secure attachment relationship, parental warmth and support, parental modeling of sympathetic emotions, parental encouragement of children’s expressions of emotion and an inductive child-rearing style most likely also contribute to consistency in sympathetic responding over time (see Eisenberg et al., 2014, for an overview of studies).

Because of the aforementioned reasons, self-reports and observations were aggregated. The mean-level differences of this aggregated measure confirmed the above mentioned depiction concerning age differences; revealing a significant increase in sympathy over the course of the study with the most increase between the first 2 years. These 2 years cover the transition from preschool to elementary school, a time that can be characterized as a critical life event for the children. The new context of socialization seems to stimulate increases not only in the development of cognitions, but also of emotions. Progress in cognitive development may, as outlined by Hoffman (2000), make children understand better what lies behind other’s feelings, thus stimulating also an increase in empathetic responses.

Furthermore, children are confronted with new expectations from parents, but also from new significant adults in their lives – the teachers. The developing relationships between elementary school teachers and children may be an important factor for the development of sympathy. As has been demonstrated elsewhere for child care teachers (Kienbaum, 2001), children are more sympathetic when they attend a classroom with a warm and supporting teacher.

Thus, the conclusion concerning the question of age-correlated development is first that *stability in sympathy is high* and secondly that there is an *increase in sympathy*, mainly during the time between the last year in child care and the first year in elementary school. The obtained effect size (omega squared) for time in the aggregated sympathy-variable can be interpreted as medium, since according to Cohen (1988, p. 286–287), values of 0.01, 0.06, and 0.14 can be used to indicate small, medium or large associations between the variables, respectively (see also Field, 2009, p. 390). The data therefore confirm the position of Hoffman (2000) who had postulated an increase of sympathy over the childhood years. But what about the rules described by Hay (1994) that should produce a decline in empathic responding, since children learn who does and who does not deserve sympathy? Maybe these rules contribute to the interindividual differences between children, since some children may hear them more frequently than others or are taught more of them than others. Thus, Hay’s theory may be more useful in explaining the emergence of interindividual differences between children, whereas Hoffman’s theory can better explain age-correlated development. A third possibility besides increase and decline had recently been expressed by Davidov et al. (2013), suggesting that empathic concern may not grow over time at all because it is an emotion, and the authors suppose that emotions do not develop like cognitions or behaviors. But are emotions and cognitions really that different? If a person feels fear, this is a prerational way of saying “this object can be dangerous to me” (Bischof, 1989). Thus, emotions and cognitions are closely related, being the two sides of a coin, whereas emotions and rationality surely have to be differentiated. The phenomenon of feeling may not change with age. This is something that cannot be taught and is part of our nature. But the intensity, the frequency, the situations in which we show our feeling or not and the actions that might follow or not, this may all change with cognitive maturation and experience. So, in sum, it makes sense that we actually found an increase in our aggregated measure of sympathy.

There are several limitations to the present study. The sample was not very large and came from one cultural subgroup: children living in Europe in a comparatively wealthy, rural environment. So the results may not be generalizable to other socioeconomic or ethnic groups. The limited number of participants also impeded the application of other ways to analyze the validity of the methods, like multitrait-multimethod-analysis (Campbell and Fiske, 1959). Further, we do not know whether the quality of the teacher’s rating might have been dependent on the type of their education. In Italy, by the time this study was conducted, part of teachers (both child care and elementary school) had a University degree, but another part started to work right after the completion of a so called pedagogical high school. Testing whether there is a relation between quality of rating and length/quality of education would be a topic for further research.

Irrespective of these constraints, the present research highlights the importance of the methods we use in our studies. The claim for longitudinal design using a multi-method multi-informant approach is not new, but rarely realized. Relying on the aggregated measure of children’s sympathy that had been derived from the observations and self-reports, we can conclude with quite high confidence that sympathy does increase during the transition from childcare to elementary school and that interindividual differences are of high stability during the childhood years.

One more question left unanswered by the data presented so far is which variables contribute to the interindividual differences between the children. The teacher-child relationships mentioned earlier are but one possibility. The child’s relationship with his or her parents (e.g., Spinrad and Stifter, 2006), his or her temperament (e.g., Eisenberg et al., 2007), the cultural context in which the children are rised (e.g., Trommsdorff et al., 2007) are but a few possibilities (see Eisenberg et al., 2006, for an overview). More research will show how this important motivator of prosocial behavior can best be promoted.

## Conflict of Interest Statement

The author declares that the research was conducted in the absence of any commercial or financial relationships that could be construed as a potential conflict of interest.

## Footnotes

1 The weights were assigned by the formula υ_ij_ = | i-j|.
